# Invasive slipper limpets *Crepidula fornicata* are hosts for sterilizing digenean parasites

**DOI:** 10.1017/S0031182022000257

**Published:** 2022-05

**Authors:** Emma A. Quinn, Jessica E. Thomas, Sophie H. Malkin, Molly-Jane Eley, Christopher J. Coates, Andrew F. Rowley

**Affiliations:** Department of Biosciences, Faculty of Science and Engineering, Swansea University, Singleton Park, Swansea SA2 8PP, Wales, UK

**Keywords:** Disease connectivity, epizootiology, invertebrate pathology, microphallids, parasitic castration

## Abstract

Invasion and spread of alien species can drive ecosystem changes, such as, the dynamics of infectious diseases. The non-native, marine gastropod *Crepidula fornicata* has become established across European coastlines over the last century, but there remains little insight into its disease carrying capacity and potential role as a source/sink of parasites. To address this knowledge gap, we surveyed limpets from two sites in South Wales, UK for signatures of disease/pathology using polymerase chain reaction-based methods (haemolymph) and histology (solid tissue). We encountered trematode-like parasites in ~1% individuals (5 out of 462). Three limpets displayed gross damage in the gonad, i.e. castration, and encysted metacercariae were found in the muscle of two other individuals. On the basis of 28S rDNA and internal transcribed spacer 2 genomic targets, we identified the gonad-infecting trematodes as members of the family Microphallidae – putative novel species related to the genus *Longiductotrema*. Earlier reports suggest that *C. fornicata* is not a host for trematode parasites in either its native or alien range but may act as a sink due to its filter feeding lifestyle. We provide clear evidence that *C. fornicata* is parasitized by at least one trematode species at two sites in Wales, UK, and likely act as a spillback or accidental host among native littorinids.

## Graphical abstract

**Figure:**
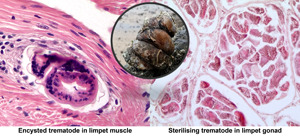

## Introduction

The American slipper limpet, *Crepidula fornicata*, is a filter feeding, sessile gastropod native to the east coast of the USA. In the late 19th century, limpets were accidently transported to the UK along with imported oysters (Blanchard, 1997, 2009). Following its arrival in Europe it was labelled as a cause of the decline in native oyster (*Ostrea edulis*) fisheries and hence referred to as an ‘oyster pest’. However, detailed analyses have shown that it has both positive and negative effects at population and ecosystem levels (e.g. de Montaudouin *et al*., 1999; Thieltges *et al*., 2006; Lown *et al*., 2021), and that its perceived responsibility for historical oyster declines in some parts of Europe may be incorrect (Hayer *et al*., 2019). On the negative side, the presence of *C. fornicata* (live or dead) increases siltation by promoting the build-up of pseudofeces and enhances sediment erodibility, resulting in damage to other filter feeders such as mussels and oysters (Grasso *et al*., 2020). Additionally, slipper limpets compete with such bivalves for space and food (e.g. Thieltges, 2005). A putative positive effect of the slipper limpet could be as a sink for digenean parasites of native molluscs (Thieltges *et al*., 2009). These authors found that incubation of blue mussels, *Mytilus edulis* with infective stages (cercariae) of the digenean, *Himasthla elongata*, in the presence of non-native slipper limpets or Pacific oysters, *Crassostrea gigas*, resulted in a reduction in levels of parasitization by these non-natives acting as alternative targets. While slipper limpets appeared to attract the cercariae of *H. elongata*, they did not become infected. Indeed, it has been questioned whether slipper limpets are compatible hosts for trematode parasites in general (Pechenik *et al*., 2001; Thieltges *et al*., 2004).

Digenean parasites of marine fauna often have complex life cycles. For instance, in the microphallid, *Microphallus similis*, the definitive hosts are sea birds (Stunkard, 1957; James, 1969). Sexual reproduction of *M. similis* only occurs in this host to yield miracidia. These penetrate the tissues of various gastropods including intertidal littorinids as the first intermediate host – invading the gonadal tissue and leading to castration. Numerous cercariae are produced that infect the second intermediate hosts, decapod crustaceans (e.g. common shore crabs, *Carcinus maenas*), where they migrate and encyst in the hepatopancreas to form metacercariae. When infected crabs are predated upon by sea birds, the life cycle is then completed (Stunkard, 1957; Blakeslee *et al*., 2015).

The aim of our study was to consider whether non-native slipper limpets can act as hosts for trematode parasitization. To achieve this, we carried out a year-long disease survey across two sites in Wales where slipper limpets have become established (Bohn *et al*., 2015). We employed a dual approach using multi-tissue (e.g. gonad, gill, muscle and heart) histopathology together with polymerase chain reaction (PCR)-based probing of putative parasites found in these hosts.

## Materials and methods

### Sampling regime

Adult *C. fornicata* were collected once a month, from two survey locations over a 12-month period, January–December 2019. Limpets (*n* = 75 per month; 1800 in total across the two sites) were collected using different methods to suit the sample locations. At the Swansea Bay (SB) site (51.570345, −3.974591) these were collected *via* dredging (⩽2.9 m below chart datum), while at the Milford Haven (MH) site (Hazelbeach; 51.7042, −4.971295) limpets were handpicked intertidally from the shoreline during low tides (for further details, see Quinn *et al*., 2021). Samples were kept in a closed circulation tank with sea water (filtered from the same site) overnight before processing the following day.

### Laboratory regime

Biometric data including shell length and width, wet weight, sex and position within the ‘stack’ were recorded. *Crepidula fornicata* routinely form stacks on substratum, in which several individuals (usually smaller males) settle and adhere on top of a single, larger female. Haemolymph (blood) was collected from each limpet by removing the solid tissues using a sterile blunt-ended probe and tweezers, and aspirating the pooled haemolymph using a sterile needle (23-gauge) attached to a syringe. Haemolymph samples (100 *μ*L) were used for DNA extractions. In a minority of cases (29 out of 1800) where animals were too small for haemolymph sampling, a small piece of foot muscle (*ca*. 20 mg) was used.

### Histopathology

Whole limpet tissue mass was submerged in Davidson's seawater fixative for 24 h at room temperature without agitation, rinsed in water and stored in 70% ethanol before further processing (see Quinn *et al*., 2020). An initial 343 limpets were selected at random from both sites over the entire sampling period, with an additional 119 limpets from Milford Haven July/August 2019 screened to determine the full extent of parasitization at this site. Post-fixing, samples were dehydrated in a graded series of ethanol, transferred to either Histoclear (National Diagnostics, Atlanta, USA) or Histochoice (Fisher, Dorset, UK) and infiltrated with a series of molten wax using a Shandon™-automated tissue processor (ThermoFisher Scientific, Altrincham, UK) prior to embedding. A series of sections was cut at 5–7 *μ*m using a rotary microtome until all major limpet tissues were present – followed by staining with Cole's haematoxylin and eosin. Slides were examined for all tissues across the different regions of the limpet. At least 3–5 slides, each containing a ribbon of sections from different regions, were routinely screened. For those limpets with parasites, further re-analysis of sections was carried out across the entire animal to determine the extent of parasite spread. Slides were photographed using an Olympus BX41 photomicroscope. Images were adjusted for colour and contrast only.

### Molecular diagnostic techniques

DNA extractions were made from haemolymph, muscle and preserved material in wax blocks. For haemolymph, 100 *μ*L samples were extracted using a Sigma Aldrich GenElute™ Blood Genomic DNA kit found in preliminary studies to give best DNA yields, using the manufacturer's guidelines. The same kit was also used for the small number of muscle samples that replaced haemolymph. For wax blocks, the area of interest (i.e. the area which contained trematode infection) was identified *via* microscopy and by reference to the histopathology. A sterile blade was used to remove excess wax from around the target area and cleaned using 70% ethanol. Wax slices/fragments were taken from the three individuals previously identified by histology as positive for trematode infection in the gonad (coded as SB64 September 2019, MH4 August 19 and MH29 August 19) as well as a control sample (no evidence of trematode infection identified *via* microscopy) from each site from the corresponding month/location (SB49 September 19 and MH24 August 19). Sample weights ranged from 3.8 to 38.7 mg (mean 21 mg). Total genomic DNA was extracted from the histology wax blocks from the trematode positive and control samples using a Qiagen QIAamp^®^ DNA FFPE tissue kit (Cat. No. 56404) and deparaffinization solution (Cat. No. 19093; Qiagen, Hilden, Germany) following the manufacturer's protocols. The extracted DNA was quantified using a Qubit^®^ dsDNA high sensitivity assay kit and fluorometer (Invitrogen, California, USA).

### PCR-based methods and amplicon sequencing

Several combinations of oligonucleotide primers were synthesized by Eurofins (Ebersberg, Germany) and deployed to identify the gonadal parasites (Table 1). PCR reactions were performed in a T100 PCR thermal cycler (BioRad Laboratories Inc., Hemel Hempstead, UK).

**Table 1.** Oligonucleotide primers tested for trematode DNA amplification

Marker	Primer name	Primer sequence (5′–3′)	Fragment size (bp)	Reference
COI	Dice1F	TTWCNTTRGATCATAAG		Moszczynska *et al*. (2009)
	Dice11R	GCWGWACHAAATTTHCGATC	580	Van Steenkiste *et al*. (2015)
	Dice14R	CCHACMRTAAACATATGATG	800	Van Steenkiste *et al*. (2015)
28S	LSU5	TAGGTCGACCCGCTGAAYTTAAGCA	1400	Jensen and Bullard (2010)
	1200R	GCATAGTTCACCATCTTTCGG		Jensen and Bullard (2010)
	28S_300F	CAAGTACCGTGAGGGAAAGTTG	600	Tkach *et al*. (2003)
	28S_ECD2	CTTGGTCCGTGTTTCAAGACGGG		Tkach *et al*. (2003)
ITS2	3S	GGTACCGGTGGATCACGTGGCTAGTG	500	Bowles *et al*. (1993)
	ITS2.2	CCTGGTTAGTTTCTTTTCCTCCGC		Cribb *et al*. (1998)
	GA1	AGAACATCGACATCTTGAAC	400	Anderson and Barker (1998)

Cytochrome c oxidase I (COI) PCR reactions and conditions were performed as per Van Steenkiste *et al*. (2015). PCR reactions were carried out in 25 *μ*L total reaction volumes containing 3.5 mm MgCl_2_, 0.2 mm dNTPs, 0.6 U Platinum^®^ *Taq* polymerase, 1× PCR buffer MgCl_2_ (Invitrogen™, ThermoFisher Scientific), 0.5 *μ*m of each primer and 5 *μ*L of template DNA. Thermocycling conditions were as follows: 1 cycle of 94°C for 2 min; 3 cycles of 94°C for 40 s, 51°C for 40 s, 72°C for 1 min; 5 ‘touchdown’ cycles of 94°C for 40 s, 50 to 46°C for 40 s (dropping 1°C per cycle), 72°C for 1 min; 35 cycles of 94°C for 40 s, 45°C for 40 s, 72°C for 1 min and a final extension at 72°C for 5 min.

For primer set LSU5/1200R, PCR reactions were performed in a 25 *μ*L total reaction volume with 1.5 mm MgCl_2_, 0.2 mm dNTPs, 0.5 U Platinum^®^ *Taq* polymerase, 1× PCR buffer MgCl_2_ (Invitrogen™, ThermoFisher Scientific), 0.4 *μ*m of each primer and 1 *μ*L of template DNA. Thermocycling conditions were: 5 min at 95°C; 35 cycles of 94°C for 30 s, 55°C for 45 s and 72°C for 2 min and 72°C for 5 min. PCR reactions for 28S_300F/28S_ECD2 were carried out in a 20 *μ*L total reaction volume with 1.5 mm MgCl_2_, 0.2 mm dNTPs, 0.5 U Platinum^®^ *Taq* polymerase, 1× PCR buffer MgCl_2_ (Invitrogen™, ThermoFisher Scientific), 0.2 *μ*m of each primer and 1 *μ*L of template DNA. Thermocycling conditions were: 3 min at 94°C; 35 cycles of 94°C for 30 s, 55°C for 30 s and 72°C for 1 min and 72°C for 2 min. PCR reactions using the 3S/internal transcribed spacer 2.2 (ITS2.2) primer set were carried out in a 20 *μ*L total reaction volume containing 2 mm MgCl_2_, 0.4 mm dNTPs, 0.6 U Platinum^®^ *Taq* polymerase, 0.5× PCR buffer MgCl_2_, 0.4 *μ*m of each primer and 1 *μ*L of template DNA. Thermocycling conditions were: 1 cycle of 95°C for 3 min, 45°C 2 min, 72°C for 90 s, 4 cycles of 95°C for 45 s, 50°C for 45 s, 72°C for 90 s; 30 cycles of 95°C for 20 s, 52°C for 20 s, 72°C for 90 s and a final extension at 72°C for 5 min (Cutmore *et al*., 2013; Hill-Spanik *et al*., 2021). For primer set GA1/ITS2.2, PCR reactions were carried out in a 25 *μ*L total reaction volume containing 1.5 mm MgCl_2_, 0.2 mm dNTPs, 0.5 U Platinum^®^ *Taq* polymerase, 1× PCR buffer MgCl_2_, 0.5 *μ*m of each primer and 3 *μ*L of template DNA. Thermocycling conditions were: 15 s initial denaturation at 94°C; 32 cycles of 94°C for 30 s, 56°C for 30 s, 68°C for 51 s and a final extension at 68°C for 3 min.

Positive PCR products producing a single band were purified using HT ExoSAP-IT™ Fast high-throughput PCR product clean-up (ThermoFisher Scientific, Altrincham, UK). Samples producing a double band and/or non-specific amplification results were purified *via* gel extraction using a QIAquick gel extraction kit (Qiagen, Hilden, Germany) following the manufacturer's protocols. Purified PCR products were sent for Sanger sequencing, in both forward and reverse directions, with Eurofins Genomics (Germany). Sequences derived from 28S rDNA and ITS2 probing for each limpet, SB64_Sept_19, MH4_Aug_19 and MH29_Aug_19, were archived in GenBank under the accession numbers OL812727–OL812729 and OL813475–OL813479, respectively.

### Phylogenetic analysis

Resolved sequences from limpets targeting the 28S rDNA and ITS2 genomic regions were queried against the GenBank nucleotide database using the basic local alignment search tool (BLAST; Altschul *et al*., 1990). A broad set of reference sequences from the Microphallidae were used to infer putative phylogenetic relationships. Sequences were inspected and trimmed manually, and independent multiple alignments were performed for both the 28S rDNA and ITS2 targets using the MUSCLE function in MEGAX (Kumar *et al*., 2018). Evolutionary reconstructions were performed using the maximum-likelihood method based on either the general time reversible model (gamma-distribution, partial deletion at 95%) for the 28S rDNA sequences or the Kimura 2-parameter model (gamma-distribution, complete deletion) for the ITS2 sequences. DNA substitution models were chosen based on the lowest Bayesian information criterion scores *via* ModelFinder. Consensus trees with the highest log-likelihood values from 1000 bootstrap re-samplings were visualized using iTOL (Letunic and Bork, 2019).

## Results

### Histopathology screening of limpets for trematodes

Histology was used as the primary screening method for the presence of trematode parasites using a reduced sample size of 462 (of the 1800 total samples) over the two sampling sites, with at least nine samples per site per month. Five limpets out of 462 showed signs of parasite presence in at least one tissue type. When considering the gonadal tissue alone, two female limpets out of 75 from Milford Haven in August (nos. 4 and 29) and one out of 75 from Swansea Bay in September (no. 64) were infected. Examination of limpets from all other months did not reveal evidence of these parasites in the gonadal tissue.

**Fig. 1.**
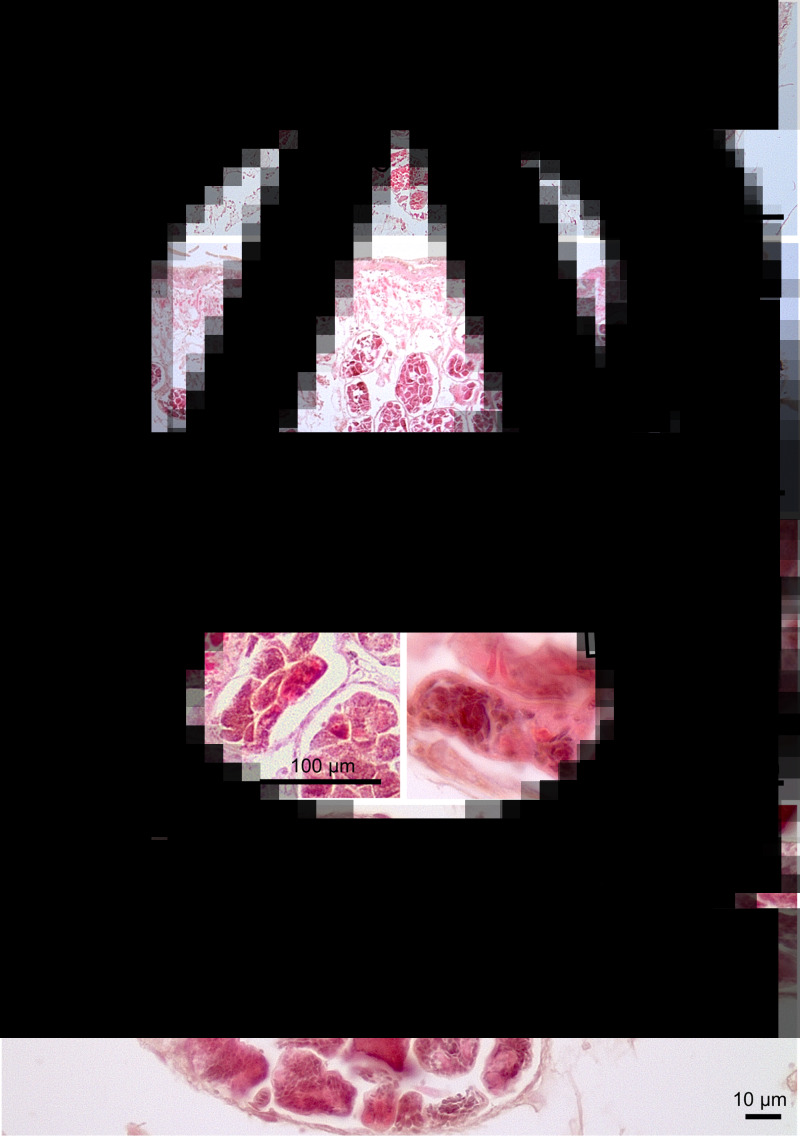
Sporocysts containing developing cercariae in a slipper limpet (no. 4) collected from Milford Haven in August 2019. (A) Low power micrograph showing the morphology of egg production in uninfected slipper limpet from August with mature, yolk-laden oocytes (Oo) and adjacent digestive gland (Dg). (B) Low power micrograph of infected slipper limpet showing replacement of gonadal tissue by parasites. (C) Remaining oocyte (Oo) around parasites in gonadal tissue. (D) High power micrograph of developing cercariae with characteristic integument (unlabelled arrow). (E) Tail regions of developing cercariae (unlabelled arrows).

The gonadal tissue in female limpets is extensive and consists of large numbers of developing oocytes containing prominent yolky cytoplasmic droplets within follicles (Fig. 1A). In those limpets with trematode parasites, both mature yolk-filled oocytes and immature yolk-less oocytes were largely absent and the spaces in which the gonadal tissue had resided were replaced by sporocysts/rediae containing large numbers of developing cercariae (Fig. 1B–E). The tail regions of these cercariae were visible in some sections (Fig. 1E). The adjoining digestive gland was infiltrated by parasites although there was no evidence of any damage to the tubules and intertubular spaces of this tissue. All three infected limpets showed similarly high levels of infection.

**Fig. 2.**
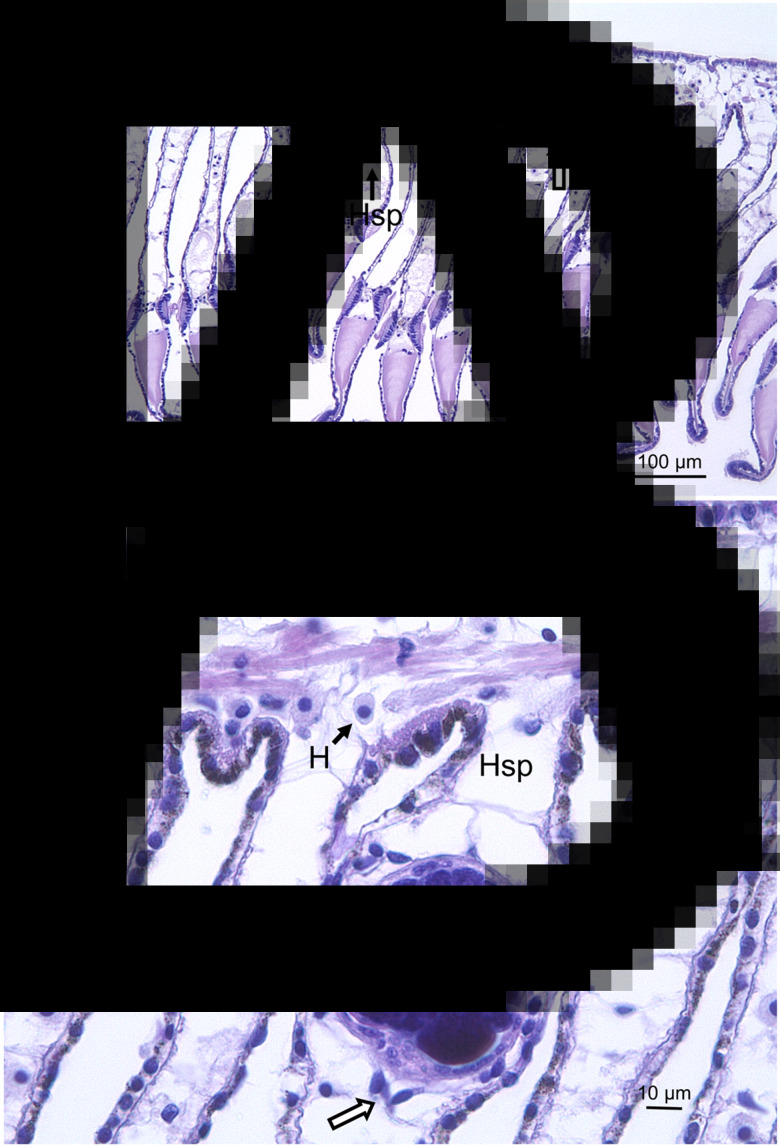
Sporocysts lodged in the gills of a slipper limpet (no. 29) from Milford Haven. (A) Low power micrograph showing sporocysts (unlabelled arrows) in the haemal space (Hsp) of gills. (B) High power micrograph of sporocyst in haemal space (Hsp) at the base of a gill filament. Note round-shaped haemocyte in circulation (H) and flattened haemocytes close to the sporocyst (unlabelled arrow).

**Fig. 3.**
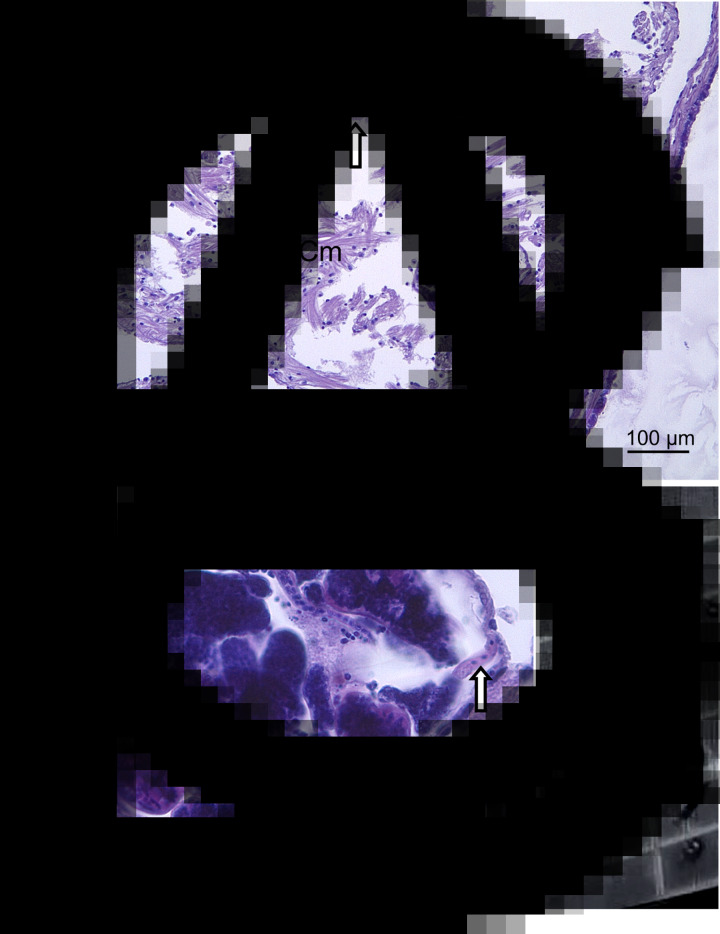
(A, B) Sporocysts (unlabelled arrows) of trematode parasite seen in the heart of an infected slipper limpet (no. 4) from Milford Haven. Cm, cardiac muscle, Sw, sporocyst wall. (C) Photograph of live swimming cercaria observed in the haemolymph of slipper limpet no. 4. Still image captured from a movie.

Evidence of spread of the sporocysts containing cercariae into other tissues *via* the haemolymph was found. In the gills, these were observed in the haemal spaces in the central region of gill filaments (Fig. 2A and B). Similarly, sporocysts were observed in the heart of one limpet (Fig. 3A and B). Examination of fresh haemolymph preparations revealed the presence of free cercariae in one of the three infected limpets (no. 4 MH August; Fig. 3C). These had single tails and were actively swimming by a combination of body extension and contraction and tail movement. In all cases there was little evidence of any extensive host reaction involving the defensive haemocytes to the presence of developing parasites.

**Fig. 4.**
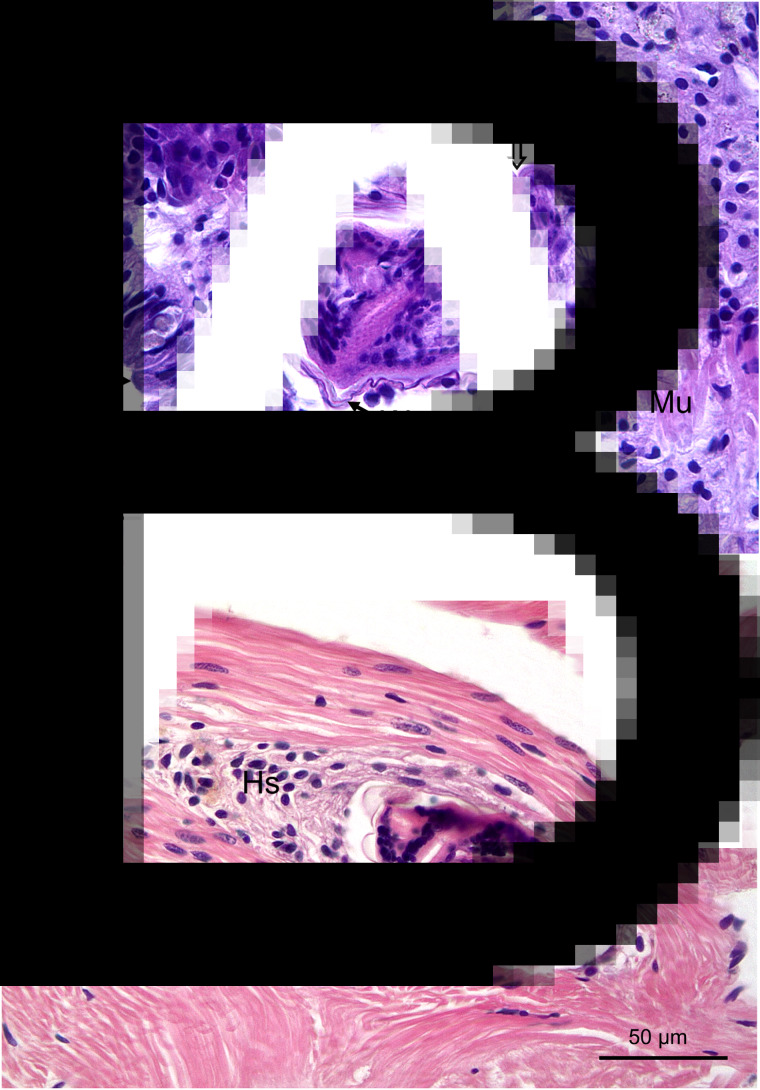
Encysted metacercariae-like unidentified trematodes in foot muscle from slipper limpets collected in August (A) and May (B) in Milford Haven. Mu, muscle; Hs, haemocyte sheath around parasite; Ep, outer epithelium; W, wall of parasite. Unlabelled arrow shows possible opening of oral region of parasite.

Two limpets from Milford Haven (no. 16 in May and no. 41 in August) were found to have unidentified metacercariae-like trematode parasites in the muscle. In both cases, these evoked a marked host defence reaction with haemocyte infiltration and ensheathment around the parasites (Fig. 4A and B). There was no evidence of any muscle breakdown around these parasites and no other tissues showed evidence of trematode infection. No similar parasites were found in muscle from limpets collected in Swansea Bay.

### PCR-based detection of trematodes in limpets

Samples identified as positive for trematode infection *via* histology were initially tested with primer sets Dice1F/Dice11R and Dice1F/Dice14R. PCR amplification of DNA in extractions from wax blocks with both COI primer sets was unsuccessful. Subsequently, the COI and 28S primer sets were tested with DNA extracted from the wax blocks and haemolymph from the same samples. A positive PCR result was obtained with each primer set from the haemolymph samples only, although a double band was observed with the upper band corresponding to the expected fragment size. All remaining haemolymph samples [i.e. all July, August and September samples from Milford Haven (*n* = 225 in total) and September samples from Swansea Bay (*n* = 75)] were tested with the 28S primer sets. Every sample, except the negative (template-free) control, generated an amplicon of the expected size on the gel. However, the fragment was smaller (~1000 bp for LSU5/1200R) than the expected size (~1400 bp for LSU5/1200R). Only the samples previously identified as showing trematode infection in the gonad showed a double band with the expected fragment size.

Additional PCR amplifications using the COI and ITS primer sets were performed with the haemolymph samples from the trematode-positive samples. Both sets of ITS primers gave a positive result and were sequenced. The Dice1F/Dice11R primer set produced a great deal of non-specific amplification and so only the Dice1F/Dice14R amplicons were sequenced. Sequences retrieved from the COI-targeting PCR were of little use, matching limpet and other molluscan DNA.

### Phylogenetic analysis of trematode ecotypes

**Fig. 5.**
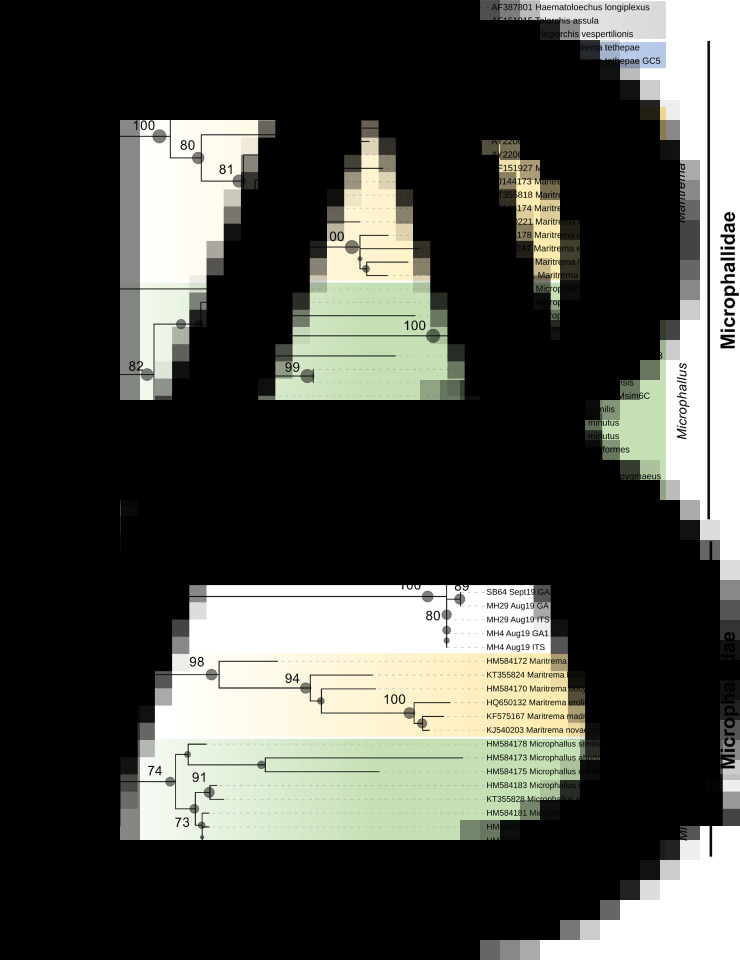
Consensus phylograms of digenean trematodes based on partial 28S rDNA (A) and ITS2 (B) genomic targets. Phylogenetic relationships were reconstructed using maximum-likelihood analysis and 1000 bootstrap re-samplings. Spheres at each node represent bootstrap support for each partition – those that received >70% are highlighted. Tree (A) is rooted using the corresponding 28S rDNA from *Haematoloechus longiplexus*, *Telorchis assula* and *Plagiorchis vespertilionis* (outgroup is coloured in grey), whereas tree (B) is unrooted. Sequences of trematodes retrieved from limpets are uncoloured (except for KC012521) and denoted by location, Swansea Bay (SB) or Milford Haven (MH). The respective scale bars indicate nucleotide substitutions per site.

BLASTn searches of the 28S rDNA sequences amplified from *C. fornicata* – SB64/Sept19 (560 bp; OL812727), MH4/Aug19 (1175 bp; OL812728) and MH29/Aug19 (1176 bp; OL812729) – retrieved consistently high matches (*e*-value = 0), 98–99% coverage and 90.2–91.5% identity, to *Microphallus minutus* (KT355823; Kudlai *et al*., 2015) and *Longiductotrema tethepae* (KX712085; Kudlai *et al*., 2016). Using a combination of oligonucleotide primers targeting the ITS2 region (Table 1), several amplicons of varying length, 432–516 bp (OL813475- OL813479), matched with *Maritrema* sp. CC-2013 (KC012521; 97–98% coverage, 96.5–97.4% identity) and *L. tethepae* (KX712087; 73–75% coverage, 85.8–87.8% identity). Independent phylogenetic analysis of 28S rDNA (Fig. 5A) and ITS2 (Fig. 5B) sequence sets using maximum-likelihood methods showed consistent topology and placement of the *C. fornicata* sequences within the monophyletic Microphallidae. Trematode sequences from all three parasitized limpets, representing both sampling sites, appeared most closely related to *Longiductotrema* isolates – 75% node support – from Moray eel, *Gymnothorax pseudothyrsoideus*, sampled around Lizard Island, Australia (Kudlai *et al*., 2016). Notably in the ITS2 tree (Fig. 5B), all trematode sequences from *C. fornicata* branched (100% support) with the *Maritrema* sp. CC-2013 sequence, a trematode observed in the closely related slipper snail *Crepipatella dilatata* from Puerto Madryn, Argentina (Gilardoni *et al*., 2011).

## Discussion

The American slipper limpet, *C. fornicata* is a highly successful species in terms of its ability to invade and establish itself in coastal and estuarine environments. Generally, it is perceived to cause damage to natural oyster (*O. edulis*) and non-native (e.g. Pacific oyster *C. gigas*) aquaculture because of its competition for space, food and enhanced siltation (Blanchard, 1997; Preston *et al*., 2020). In parts of northern Europe, it reaches high density in both intertidal and subtidal areas (Blanchard, 1997; Thieltges *et al*., 2004; Bohn *et al*., 2012). The success of non-native populations of slipper limpets has been attributed to several factors including high fecundity, where females can produce several batches of eggs annually and their brooding ability of fertilized eggs that reduces larval mortality (Pechenik *et al*., 2017) together with an extended reproductive period (e.g. Ricard *et al*., 2006; Bohn *et al*., 2012). While cold winters appear to limit its current range within Europe (Thieltges *et al*., 2004), climate change and rising temperatures in coastal waters may lessen this (Valdizan *et al*., 2011). Soon, this species (and perhaps its pathobionts) may migrate into northern latitudes in Europe. Conversely, projected higher temperatures in the summer months may become detrimental to those slipper limpets found in the intertidal area (Pechenik *et al*., 2020).

There have been very few studies on parasites and pathogens of slipper limpets, both in native and non-native populations, except for one report of their colonization by the shell-boring sponge, *Cliona celata* but even this was found to have few negative effects on the host (Le Cam and Viard, 2011). As part of our yearlong survey of non-native slipper limpets in these two locations in south and southwest Wales, we sought to address this by carrying out the first systematic disease survey. Interestingly, we noted punctate holes in the shells of 2–3 limpets (out of 1800, unpublished observations), but the cause could not be attributed to shell-boring sponges as stated above or by molluscan radulae. In terms of bacterial load in the haemolymph (a potential marker of bacterial sepsis), Quinn *et al*. (2021) found that *C. fornicata* at the same sites studied here were largely free from bacterial diseases and importantly were not reservoirs for either mollusc (e.g. *Vibrio aestuarianus*) or human (*Vibrio parahaemolyticus* and *Vibrio vulnificus*) pathogenic vibrios. In the current study, the prevalence of trematode parasitization was small (~1%) suggesting that it has a limited impact on the fecundity of slipper limpet populations and on other hosts of these parasites including crabs and sea birds. Furthermore, successful metacercarial infections are rare in *C. fornicata*, but limpets could still attract cercariae that fail to establish an infection. On the basis of our observations, we cannot exclude the possibility that the common slipper limpet is acting as a sink for cercarial development that may relieve ‘pressure’ on other gastropod and/or bivalve intermediate hosts. Filter feeders in general – *C. fornicata* is a rare gastropod example – have strong and efficient cercarial removal capacity (e.g. Burge *et al*., 2016). We consider that the trematode infections seen in our study are probably accidental (or spillback) from invasive miracidia and possibly for cercarial stages in the parasite's life cycle. The principal primary intermediate hosts in both Swansea Bay and Milford Haven could be other littorinids, *Littorina* spp. that abound at both locations and have been reported to be hosts for a range of microphallids including *M. similis* and *Microphallus pygmaeus* in the UK (James, 1969; Bojko *et al*., 2017). However, this would depend on the susceptibility of littorinids to this potentially novel microphallid, which until now has not been explored.

Through phylogenetic analyses, we show that the gonad-infecting trematode of slipper limpets is closely related to a recently discovered microphallid, *L. tethepae* found in its definitive host, the highfin Moray eel (*G. pseudothyrsoideus*) from the northern Great Barrier Reef, Australia (Kudlai *et al*., 2016). The metacercariae of *L. tethepae* were also found in a grapsid crab, *Grapsus albolineatus* as a second intermediate host but the presence of earlier life history stages in molluscs was not reported. To our knowledge, there have been no reports of *L. tethepae* outside of Australia. Metacercariae of an unidentified species of *Longiductotrema* have also been found in crabs (*Xantho exaratus*) in the Arabian Gulf (Abdul-Salam *et al*., 1997), but these lack molecular characterization. While our phylogenetic analyses strongly support similarity to *L. tethepae*, they also suggest that the parasite of slipper limpets may be novel at the species and/or genus level; however, without detailed morphometrics of all the life history stages of the parasite, it is premature to speculate further.

One surprising observation of our study is the lack of early stages of parasitization in that all the infected animals found were essentially almost fully castrated such that their reproductive ability was highly compromised. Early stages of parasitization were not observed in the slipper limpets from August in Milford Haven and September in Swansea Bay, and in other months of the year. The finding of parasitization in a single month at each site was also unexpected. Our study contrasts with the only other report of trematode parasitization in other species of slipper limpets where a putative *Maritrema* sp. was found at high prevalence (*ca*. 33%) in native *C. dilatata* from Punta Cuevas, Argentina (Gilardoni *et al*., 2011, 2012). Unlike our study, they found infected limpets throughout the period of sampling with all seasons harbouring infected *C. dilatata* and with a peak in parasite prevalence in winter. They also observed variation in levels of severity of infections on egg development of the host from their histology (Gilardoni *et al*., 2011). *Crepipatella dilatata* is native to Argentina and Chile and similar to *C. fornicata* it has extended its range into Europe. Here, it was first observed in northern Spain in 2005 and its identification was later confirmed by Collin *et al*. (2009). It is thought that there may have been multiple introductions of *C. dilatata* into Europe with imports of live mussels (*Mytilus galloprovincialis*) from Chile (Richter *et al*., 2018). Unfortunately, to our knowledge, there are no published reports of the trematode infections in this non-native species in its new location in Spain that would shed light on whether these parasites found in native limpets survive the transition into the host's non-native range.

The arrival and colonization of marine invaders can have profound effects on existing host–parasite ecology. Goedknegt *et al*. (2016) recognized six direct and indirect effects on both invader and native species as well as on their existing parasites. A common effect is a reduction or loss of parasites in non-native invaders. Loss of parasites in invading species has been widely reported in both vertebrate and invertebrate non-natives (Torchin *et al*., 2003; Blakeslee *et al*., 2012; see review by Blakeslee *et al*., 2013). The second scenario of Goedknegt *et al*. (2016) occurs when non-natives encounter existing infections/parasites allowing these to acquire new hosts and thereby interrupting the intimate balance between these. The origin of this novel microphallid in *C. fornicata* at the two sites studied is unclear (i.e. whether it was acquired or brought in with the original invasion), and there remains a paucity of information on the trematode parasites of slipper limpets in both their native and extended locations.

## Concluding remarks

Despite earlier studies finding little direct evidence of digenean parasites in American slipper limpets *C. fornicata* (Pechenik *et al*., 2001; Thieltges *et al*., 2004), we observed low levels of parasitization by a potentially novel species of microphallid at two sites in South Wales, UK (restricted to 2 months of the year). Hence, the failure of others to observe such events may have been due to their limited surveys both in terms of numbers of individuals examined and the timescale of these. The low prevalence of parasitization suggests that *C. fornicata* is an accidental host. We also consider these infections are likely to have few ecological consequences for native gastropods acting as primary intermediate hosts in the same locations. Overall, the lack of microbial (Quinn *et al*., 2021) and macrobial diseases found in *C. fornicata* at these two sites may provide an additional explanation of their success in the establishment of high-density populations in European coastal waters over the last century.

## Data Availability

All sequence data have been deposited in GenBank under the accession numbers OL812727–OL812729 (28S rRNA) and OL813475–OL813479 (ITS2).
